# Supplementary material for: Content-rich biological network constructed by mining PubMed abstracts
Source: BMC Bioinformatics. 2004 Oct 8;5:147. doi: 10.1186/1471-2105-5-147 (PMC528731; doi:10.1186/1471-2105-5-147)
Supplement: Additional File 2 — The original results of the above study (non-essential files are deleted to keep the file size under the limit set by BMC bioinformatics). [file 1471-2105-5-147-S2.bz2 › chilibotAdditionalFile2/dip05/524ID9636146E10829/html/SP100_CBX5.html]

 


 **SP100** and **CBX5** 
  
Found 5 abstracts in PubMed, retrieved 05.  
 

 What does Google say? 
 PDF only 
| .edu only 

---

**Interactive relationship** (e.g. stimulation, inhibition, etc)

**Stimulatory relationship**- Finally, in vitro experiments indicate that SUMO modification of  **SP100**  enhances the stability of  **SP100**  HP1  [ **CBX5** ]  complexes.  Ref: 11313457 Mol Cell Biol, 2001
- Both HP1  [ **CBX5** ]  and  **SP100**  HMG concentrate in the PML  **SP100**  NBs, and overexpression of  **SP100**  leads to enhanced accumulation of endogenous HP1  [ **CBX5** ]  in these structures.  Ref: 9636146 Proc Natl Acad Sci U S A, 1998
**Neutral relationship**- In exploring the functional relationships between NBs and chromatin, we have shown previously that  **SP100**  interacts with members of the HP1  [ **CBX5** ]  family of nonhistone chromosomal proteins.  Ref: 11313457 Mol Cell Biol, 2001
- Here, we show that  **SP100**  binds to members of the heterochromatin protein 1 HP1  [ **CBX5** ]  families of non histone chromosomal proteins.  Ref: 9636146 Proc Natl Acad Sci U S A, 1998
- Interaction of  **SP100**  with HP1  [ **CBX5** ]  proteins a link between the promyelocytic leukemia associated nuclear bodies and the chromatin compartment.  Ref: 9636146 Proc Natl Acad Sci U S A, 1998
- In this report, we have reconstituted and characterized the HP1  [ **CBX5** ]  KAP 1 interaction using purified proteins and have compared KAP 1 to three other known HP1  [ **CBX5** ]  binding proteins  **SP100** , lamin B receptor LBR, and the p150 subunit from chromatin assembly factor CAF 1 p150.  Ref: 10938122 Mol Cell Biol, 2000
- The lysine residue of the  **Sp100**  protein, to which SUMO 1 is covalently linked, was mapped within and may therefore modulate the previously described HP1  [ **CBX5** ]  protein binding site.  Ref: 10212234 J Biol Chem, 1999

**Non-interactive relationship** (e.g. studied together, co-existance, homology, etc.)

- When bound to a promoter,  **SP100** ,  **SP100**  HMG and HP1  [ **CBX5** ]  behave as transcriptional repressors in transfected mammalian cells.  Ref: 9636146 Proc Natl Acad Sci U S A, 1998
